# Evaluating a Social Robot Reception Service in an Italian Geriatric Hospital (HOSPER Study): Protocol for a Usability Study

**DOI:** 10.2196/81395

**Published:** 2026-02-26

**Authors:** Giulio Amabili, Arianna Sgolastra, Arianna Margaritini, Federico Barbarossa, Elvira Maranesi, Giacomo Cucchieri, Lidia Pascucci, Letizia Ferrara, Roberta Bevilacqua

**Affiliations:** 1 IRCCS INRCA (Istituto Nazionale di Ricovero e Cura per Anziani), Via Santa Margherita 5, Ancona, 60124, Italy, 39 0718004767; 2 Local Health Agency of Ancona, Ancona, Italy

**Keywords:** social robots, hospital setting, reception service, human-robot interaction, HRI, older adults, usability, user experience, user-centered design

## Abstract

**Background:**

Humanoid robots are being used more frequently in health care: in geriatrics and pediatrics, robots such as “Pepper” and “Nao” have been shown to enhance communication, emotional well-being, and patient engagement. During the pandemic, their role expanded, for example, to include remote monitoring and reducing the risk of infection. Given these applications, our study examines the integration of 2 advanced humanoid robots, Pepper and Nao, within the reception area of a geriatric hospital.

**Objective:**

The study aims to develop a reception service directed to enhance the quality of the IRCCS Istituto Nazionale di Riposo e Cura per Anziani (INRCA), Ancona, Italy. The primary end point of the study is the assessment of the perceived quality of an innovative reception service. Second, the study aims at evaluating the usability, human-robot interaction, user experience, and technical performance of the solution.

**Methods:**

The HOSPER (analysis of the quality of reception services using robotic platforms, Nao and Pepper) study is a feasibility pilot project coordinated by IRCCS INRCA. During the trial, 200 older adults using services at IRCCS INRCA in Ancona will be enrolled. Two humanoid robots, Nao and Pepper, which are already available at the hospital, will be used to support reception and orientation services. Data collection will include general demographics, cognitive and functional status, usability measures, perceived service quality, and human-robot interaction indicators.

**Results:**

The recruitment and data collection started in January 2025 and will be concluded by December 2025. Results regarding the feasibility of a robotic-based reception service in a geriatric hospital will be published in 2027.

**Conclusions:**

The HOSPER study aims to assess the feasibility of an innovative robot-mediated reception service in a geriatric hospital, with specific focus on recruitment feasibility, completion rates, user acceptability, and perceived service quality. In fact, the usability and user experience evaluation will demonstrate the feasibility of the approach and provide indications for future developments and improvements.

## Introduction

### Background

Hospital reception and orientation represent critical first touchpoints in the patient experience [1-3]. In geriatric settings, the process of navigating an unfamiliar hospital environment to reach the correct department or exam location is often associated with anxiety, confusion, and poor first impressions of care [4,5]. These challenges are particularly acute for older adults, who may experience navigation difficulties due to cognitive or sensory changes, physical fatigue, or limited familiarity with institutional settings [6,7]. Current human-staffed reception services, while important, are often resource-constrained and may not fully address the emotional and relational dimensions of a welcoming care experience [3,8]. In recent years, social and socially assistive robots (SAR)—autonomous machines with humanoid features designed to interact empathetically with humans—have demonstrated promise both in health care settings and in interactions with older people [9,10]. Systematic reviews confirm the effectiveness of SAR in maintaining cognitive and physical functions, promoting sociability and emotional support, and reducing feelings of loneliness and anxiety among older adults [11,12]. These robots have been successfully deployed across diverse health care contexts, from therapeutic interventions with children and individuals with autism to supportive roles in geriatric care facilities [13-15]. Prior work highlights that SAR can engage older adults in meaningful interactions, improve mood and cooperation during care activities, and enhance perceived quality of care experiences [16,17]. A growing body of evidence suggests that robots possess specific affordances for the reception and orientation context: they can provide consistent, patient, and nonjudgmental guidance; they can communicate in multiple languages and adapt their communication style to individual needs; and their embodied, interactive presence may reduce anxiety and foster a sense of welcome that improves the initial hospital experience [18-20]. On this basis, we hypothesize that deploying SAR as reception agents can improve older adults’ perceived quality of the reception and orientation experience, as well as the usability and acceptability of the service. However, current research on social robots and SAR in health care focuses on therapeutic, rehabilitative, or patient care activities. The application of SAR specifically to hospital reception and orientation services—particularly in the Italian health care context—remains understudied. Thus, a feasibility pilot is warranted to assess whether this innovation can be successfully integrated into routine hospital operations and whether it meaningfully enhances older adults’ experiences during intake.

### Goal of the Study

The HOSPER (analysis of the quality of reception services using robotic platforms, Nao and Pepper) study aims to assess the feasibility of assigning receptionist duties in an Italian geriatric hospital to two widely used SAR in research and health care settings, Nao and Pepper (both developed by SoftBank Robotics). These robots were selected because they offer multimodal interaction capabilities (voice, gesture, and visual interface), are highly customizable, and are already available at the IRCCS INRCA (Istituto Nazionale di Riposo e Cura per Anziani) hospital. Thus, this protocol focuses on improving the perceived quality of the reception and orientation experience for older adults, rather than directly measuring navigation performance (eg, time or errors). Therefore, the primary outcome is perceived service quality, with secondary outcomes capturing usability, human-robot interaction (HRI) quality, and emotional responses during robot-mediated guidance.

## Methods

### Study Design

The HOSPER study is a feasibility pilot study coordinated by IRCCS INRCA. Its aim is to assess the perceived quality of an innovative reception service through the introduction of 2 robotic platforms designated to provide information on how to reach the location where the person will receive the service of interest.

**Figure 1.**
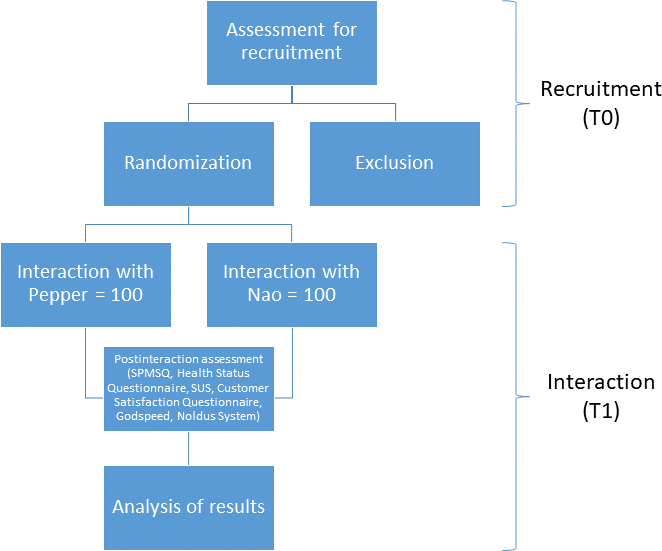
The study design. SPMSQ: Short Portable Mental Status Questionnaire; SUS: system usability scale.

During the trial period, 200 older adults referred to IRCCS INRCA in Ancona will be enrolled. Figure 1 shows the study design, which consists of 2 different phases: recruitment (T0) and interaction (T1).

A total of 200 older adults will be recruited for this single-center feasibility pilot with a pragmatic quasi-experimental design. For logistical reasons, entire days will be dedicated to either Nao or Pepper. Participants will be equally allocated to interact with Nao or Pepper based on alternating study days and a predetermined quota system: once 100 participants have interacted with one robot, all subsequent participants will use the other robot until a total of 100 participants are reached for each. The allocation schedule will be predetermined and documented to ensure transparency.

### Participants

The inclusion and exclusion criteria are reported in Textbox 1.

Textbox 1. **Inclusion and exclusion criteria for patients.**

**Inclusion criteria**

- Aged 65 years and older
- Capacity to consent
- Possession of full cognitive abilities (Short Portable Mental Status Questionnaire=0)

**Exclusion criteria**

- Failure to meet the inclusion criteria
- Concomitant participation in other interventional studies based on human-robot interaction
- Lack of written informed consent
- Pacemaker wearer
- Significant visual or hearing impairment

### Equipment

**Figure 2.**
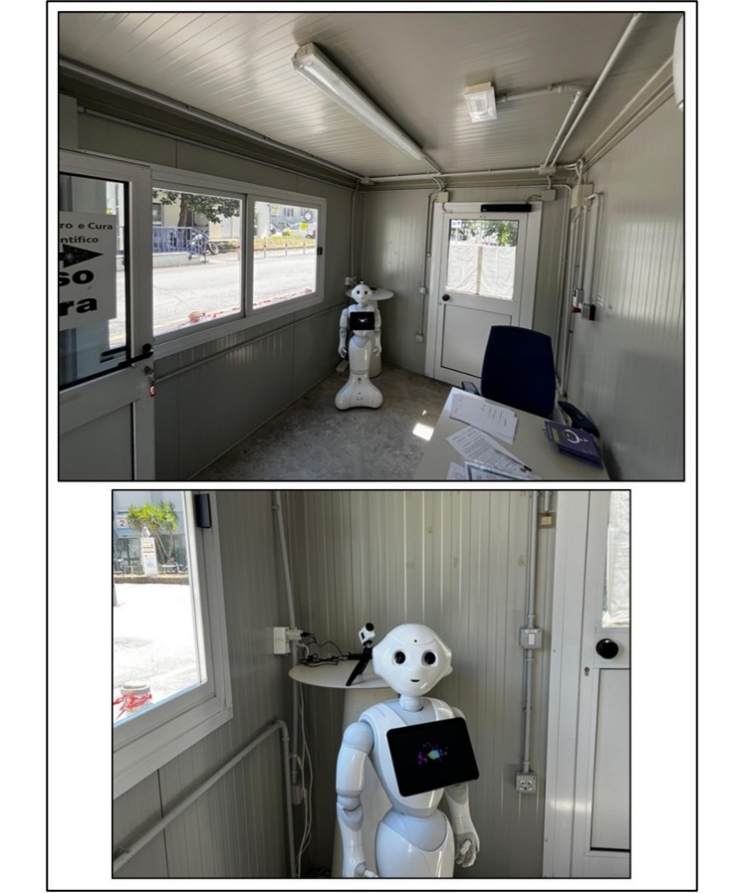
The experimental setting. (A) Dedicated area for the study with 1 robot (Pepper). (B) Focus on the experimental setting with a high-definition camera to record the human-robot interaction.

For this study, 2 humanoid social robots already available at IRCCS INRCA will be used: NAO v6 (58 cm in height) and Pepper (120 cm in height), both developed by SoftBank Robotics. The HOSPER applications for both platforms were developed using the Choregraphe software (Nao v2.8.6.23 and Pepper: v2.5.10.7; SoftBank Robotics), with standardized interaction flows programmed via “choice” objects handling up to 30 ward inputs. Interactions follow identical scripted sequences: they are user-initiated by the keyword pronunciation (HOSPER), followed by a fixed welcome prompt, speech recognition (ie, desired ward to reach), path directions provided by the robot (during this passage Pepper also shows a visual map on the tablet), repetition on request (≤40% understanding), and standardized closure. Detailed technical development is reported in Amabili et al [21]. Figure 2 shows the dedicated area and one of the robots. This is a closed box in which the robots will be located. Figure 2A shows the experimental setting, while Figure 2B focuses on the experimental setting with the high-definition camera that will record HRI.

### Outcomes

#### Overview

The primary end point of the study is the assessment of the perceived quality of an innovative reception service, as measured by the Customer Satisfaction Questionnaire.

The secondary end points are (1) assessment of the usability of 2 robotic platforms by older adults for hospital reception, as measured by the system usability scale (SUS) [22]; (2) analysis of HRI using the Godspeed Questionnaire [23]; (3) dynamic analysis of the interaction and user experience using the Noldus system [24]; and (4) analysis of robot logs to assess technical performance.

As this is a feasibility study, these outcomes will be interpreted descriptively. For the primary end point, we will examine mean scores on the Customer Satisfaction Questionnaire and the proportion of participants reporting medium-to-high satisfaction levels to judge whether the robot-mediated reception service is acceptable to older adults. For the secondary end points, the distributions of SUS, Godspeed Questionnaire scores, and Noldus-derived emotional indicators will be used to evaluate whether usability, HRI quality, and emotional responses are compatible with moving towards a larger-scale trial. In addition to these measures, the study will use standard feasibility indicators, including recruitment rate, the proportion of participants who complete the interaction and postinteraction assessments, and completeness of data collected at T0 and T1. These indicators will be summarized descriptively to judge whether the protocol is feasible in routine practice. Finally, qualitative notes on technical performance (eg, malfunctions, interruptions, or need for operator intervention) will be collected throughout the study both during the sessions and through the analysis of video recordings to inform the system-level feasibility of the robot-mediated reception service and guide future updates of the service.

While outcomes focused on usability and HRI emphasize validated patient-reported measures, triangulation is provided through existing data sources: Noldus video analysis for objective emotional and behavioral indicators, and robot logs capturing interaction duration, comprehension rates, and repetition requests as objective efficiency metrics. In fact, these complementary data sources mitigate self-report limitations while remaining within the approved protocol scope.

Finally, as this is a feasibility pilot, a priori success criteria are defined. The study will be considered feasible if: (1) at least 70% of eligible older adults approached agree to participate; (2) at least 70% of enrolled participants complete the interaction with the assigned robot and the postinteraction assessment; and (3) questionnaire data are complete for at least 80% of enrolled participants. These thresholds are intended to inform the design and sample size of a subsequent definitive trial rather than to support hypothesis testing.

#### Sociodemographic Questionnaire

This is a simple tool used to collect key personal data from study participants. The information collected includes date of birth (dd/mm/yy), sex (male, female, or intersex), current marital status (married, cohabiting, separated, divorced, single, widowed, and do not know or refuse to answer), level of education (no education, primary education, secondary education, tertiary education, and do not know or refuse), total years of education and current employment status (retired, full-time employment, part-time employment, unemployed, working from home, do not know, and refuse), details of last job, current source of income (work, pension, annuity, help from relatives, social security benefits, other, do not know, and refuse), and possible cohabitants (none, spouse or partner, children, grandchildren, sons-in-law or daughters-in-law, brothers or sisters, mother or father, assistant, others, and refuse to answer).

#### The Short Portable Mental Status Questionnaire or the Pfeiffer Test

This is a tool designed to assess cognitive impairment in older patients [25]. This tool was designed to provide health care professionals working with older patients with an effective and simple method of assessing cognitive impairment. The Short Portable Mental Status Questionnaire is a short questionnaire consisting of 10 questions investigating certain aspects of cognitive ability: 7 on orientation (spatial, temporal, personal, and environmental), 2 on long-term memory (telephone number or address and mother’s maiden name), and 1 on concentration (serial subtraction). Questions should be asked in the order they appear in the protocol, and a score of 0 is assigned for each unanswered or incorrect question, or 1 for each correct answer. The maximum possible score ranges from 0 (indicating maximum cognitive impairment) to 10 (indicating no cognitive impairment). A correction must be applied to the obtained score based on the participant’s level of education: if the number of errors is 0, no correction is made for the patient’s level of education; if the patient’s level of education is less than 8 years, subtract 1 from the raw score; if the patient’s level of education is 8 years, the raw score is not modified; and (4) if the patient’s level of education is more than 8 years, add 1 point to the raw score.

The results are interpreted based on the number of errors made: 0 to 2 errors (cognitive functions intact), 3 to 4 errors (indicative of mild cognitive decline), 5 to 7 errors (indicative of moderate cognitive decline), and 8 to 10 errors (severe cognitive decline).

This quick tool helps to define the presence and intensity (mild, moderate, and severe) of cognitive disorders of organic origin in older patients. It can be used by medical staff and other healthcare professionals in any setting. It can also be used with blind people.

#### Health Status Questionnaire

As well as asking for the participant’s age, the questionnaire uses dichotomous questions and scales ranging from 1 to 5 to assess their visual and hearing abilities. For example, it asks whether they can read a book or watch television with adequate lighting, with or without lenses, and whether they can follow a conversation or use the telephone, even with a hearing aid. The user will assess their own sense of sight and hearing, rather than the researcher.

#### SUS Tool

The SUS is a reliable tool for measuring usability. It consists of a 10-item questionnaire in which respondents are asked to indicate their level of agreement or disagreement with a series of statements using a 5-point scale ranging from “Strongly Agree” to “Strongly Disagree.” The SUS allows for the evaluation of a wide variety of products and services, including hardware, software, mobile devices, websites, and applications. The SUS is easy to administer and can produce reliable results in small samples, effectively distinguishing between usable and unusable systems.

#### Customer Satisfaction Questionnaire

This tool is designed to assess user satisfaction after using the 2 robotic platforms. Satisfaction is assessed by quantifying various variables, such as: the attention received from the robot in terms of accuracy and courtesy (scoring from 1=“not at all satisfied” to 7=“very satisfied”), the clarity and completeness of the information received (scoring from 1=“not at all satisfied” to 7=“very satisfied”), respect for personal privacy (scoring from 1=“not at all satisfied” to 7=“very satisfied”), overall satisfaction with the service offered (scoring from 1=“not at all satisfied” to 7=“very satisfied”), and whether they would recommend our service to others (scoring from 1=“absolutely not” to 7=“definitely yes”).

#### Godspeed

It allows users to quantify certain characteristics of the platform perceived during use, using a score ranging from 1 to 5 for each item. In particular, it refers to anthropomorphism, animation, friendliness, intelligence, and perceived safety.

### Procedure

#### Recruitment (T0)

The robots will be placed in a dedicated area (a closed box) at the hospital entrance. Only one user will be allowed in at a time. A researcher will stop users at the hospital entrance and invite them to participate in the study. Participants must confirm their willingness to participate in writing. After signing the consent forms, the technology will be assigned to them in a randomized alternating manner (ABAB) according to the order of inclusion of participants. Then, the researcher will collect general information about the patient and administer the cognitive scale to verify that the user meets the inclusion criteria.

#### Interaction With the Robot (T1)

The user, who has been deemed suitable and agreed to participate, starts the interaction with the robot assigned. In both cases, the researcher will suggest touching the robot’s head to initiate the interaction. From this point onwards, the researcher will not intervene in the interaction between the user and the robot, and the user will be free to interact with the robot and to stop the interaction at any time. When an explicit support request is made by the user, the researcher can intervene a maximum of 3 times, in line with good practice in experiments involving HRI analysis. At this point, the robot greets the user and asks how they can be helped, using the phrase “Good morning and welcome to INRCA. I’m (robot’s name) and I’m here to provide useful information to help you reach your desired destination within the hospital. Which department do you need to reach, or which exam do you need to take? Please, answer by saying only the name of the department or the exam as soon as my eyes turn blue.” At this point, the user responds, and the robot replies in one of two ways:

1. If it does not understand the request (or if the request is outside the robot’s knowledge), it invites the user to rephrase it and they return to the previous point; and
2. If it understands the request, it explains the path that the user should follow. In the case of Pepper, this path will also be shown on a display with photographic materials integrated with directional arrows.

After 1 successful interaction or 2 failed attempts, the robot asks the user if it has provided all the necessary information. If so, the robot says goodbye to the user as follows: “Thank you for participating. Have a nice day.” Otherwise, the interaction resumes from the point where the robot asks for the department to be reached or the test to be performed.

The researcher suggests that the user should touch the robot’s head to stop the session only in the case of an interaction loop or if the user is willing to stop the interaction.

All interactions will be recorded on video using a GoPro camera placed behind the robot. The robot will be placed in a dedicated area, and only one user will be permitted at a time to ensure that no data is collected about “unaware individuals.” These videos will be used for postanalysis to assess the emotional aspect of the interaction using the FaceReader system of Noldus in the “Youse” Usability Lab, which is located within the library of the Institute.

#### Postinteraction Assessment

A brief interview will be administered after the interaction to assess satisfaction with the service. The SUS scale will be used to measure usability grade and the HRI scale will be used to assess HRI.

### Statistical Analysis

Sample size determination followed feasibility study conventions rather than power calculations for confirmatory testing. Previous studies evaluating patient satisfaction with novel health care service innovations enrolled 124 to 177 participants [26,27]. To provide robust descriptive data across both feasibility indicators (recruitment, completion, and acceptability) and the balanced quasi-experimental design (n=100 per robot), the HOSPER study targets 200 participants (100 Nao and 100 Pepper). This sample size supports reliable estimation of prevalence estimates (eg, satisfaction proportions) while remaining practical for a single-center pilot. In the case that the statistical analysis reveals a positive trend, a new protocol will be proposed with the aim of conducting a trial.

Continuous variables will be reported as mean and SD, or median and IQR, depending on their distribution (as assessed using the Kolmogorov-Smirnov). Categorical variables will be expressed as absolute numbers and percentages.

Descriptive statistical analysis will be conducted on quantitative data with SPSS (Statistical Package for the Social Sciences; IBM Corp) and RStudio (Posit PBC). The robot’s logs (computer records) will be extracted in order to make a technical assessment of the objective parameters of HRI: dialogue comprehension ability, questions asked, answers given, dialogue duration, and so on.

Moreover, HRI will be analyzed using the Noldus system, considered the gold standard for behavioral analysis. Specifically, FaceReader and Observer (Noldus Information Technology) will be used to analyze audio-video recording of interactions. FaceReader uses facial analysis to identify the Ekman 7 emotions (neutrality, happiness, anger, sadness, disgust, fear, and surprise) in real-time [28] and summarize emotional valence distributions. Instead, the Observer enables the analysis of the interaction quantitatively and qualitatively by manually inserting labels to code the user’s emotional state and behaviors. Results will describe emotional and behavioral patterns during robot interactions to complement self-reported questionnaires. These analyses can be integrated and stacked with the Noldus software, enabling correlations to be found between the robot’s actions and the user’s behavior. This observational approach is useful for describing the dynamics of interaction and assessing the user’s experience.

Finally, the comparison between groups (Nao vs Pepper) will be carried out with a 2-tailed *t* test or the Mann-Whitney *U* test and the Fisher exact test considering the variables typology and distribution. As this is a feasibility study, these comparisons are descriptive only and not powered for formal inference. Linear or logistic regression models may be used exploratorily to examine potential confounders, but results will not support causal conclusions.

Due to the enrollment of the older population, despite the short duration of the testing and the presence of a researcher, it could happen to have missing data. Missing data will be reported by variable and time point. Moreover, available case analysis will be used for descriptive summaries, with complete-case denominators for primary outcome and feasibility indicators.

### Risks and Benefits Analysis

This study involves risks and benefits for participants. Expected benefits include the possibility that participants may feel more supported during reception, thanks to standardized information and dedicated guidance from the robots. Risks are minimal, but not absent. There is a risk that older people will stop interacting with the robots (eg, because they dislike Pepper and Nao or because they are afraid to interact with 2 humanoid robots). If this were to happen, the session would be interrupted and concluded immediately.

As the presence of the 2 robots could cause distress, participants will interact with them in an environment that guarantees an adequate level of privacy during the interaction.

To protect the security of participants, the following measures have been adopted:

1. Placement of robots within a dedicated structure that allows private data collection without external interference and ensures a human-robot interaction that respects user privacy and security;
2. The robots will function in plug-in mode to avoid service interruption and to limit their free movements. They will remain static, although they are free to gesticulate and move their heads, as these are essential aspects of empathetic interaction;
3. Clear and complete information about the appropriate use of the robots (eg, they must not lean on the devices or make any movements that could destabilize them);
4. The researcher will be present in the study area throughout the interactive session to adequately train participants on the use of robots and provide assistance in case of difficulties or problems, without impacting interaction;
5. All phases will also be illustrated with easily interpretable iconographic material to guide the researcher.

Despite the precautions described above, adverse events may still occur: (1) participants will be asked not to touch or disconnect the robot in case of problems or malfunctions; (2) participants should call the researcher if they experience any difficulties; and (3) when called by participants, the researcher will ensure that no damage has been caused to anyone or to the robots.

Although risks are expected to be low, they are not negligible. Some older adults may experience temporary fear, embarrassment, frustration, or discomfort when interacting with humanoid robots, or may decide to interrupt the interaction. To manage these risks, a researcher (either a psychologist or a physician) will be present throughout the sessions, participants will be reminded that they can stop at any time without consequences for their care, and any signs of distress will lead to immediate interruption of the interaction. A further potential risk is overattachment to or over-reliance on the robots. This will be mitigated by providing clear information that the robots are tools to support reception only, by limiting the duration of interactions, and by ensuring that human staff remain available at all times for conventional reception and orientation. Overall, the controlled environment, explicit right to withdraw, and continuous monitoring by trained researchers are expected to keep psychological risks within acceptable limits for a feasibility pilot.

Finally, if participants request longer interaction with these devices at the end of the study, they will be asked to participate in similar studies to ensure continuity in the use of the technological devices used.

### Ethical Considerations

#### Ethical Approval

The study was approved by the Marche Territorial Ethics Committee (CET MARCHE) and registered under the protocol 42021 at INRCA on December 16, 2024. The study protocol was recorded in ClinicalTrials.gov (NCT07002411). The principles of the Declaration of Helsinki and Good Clinical Practice guidelines were adhered to.

#### Data Management

All participants in this study will provide written informed consent. Throughout all procedures, including screening, recruitment, testing, evaluation, and dissemination, the project is committed to maintaining the anonymity and confidentiality of participants. Procedures for data collection, use, and storage comply with national legislation, the General Data Protection Regulation (GDPR) and the European Union’s Data Protection Impact Assessment (DPIA), including participants’ rights to access their data, to be informed, to withdraw consent, and to request data deletion. Data will be collected in accordance with the principle of data minimization, that is, only the personal information that is directly relevant and necessary for achieving the specific objectives of the testing and evaluation work packages will be collected. Data entry will be performed using dedicated software that includes checks and validations to reduce input errors. All screening data will be deleted upon project completion. During testing procedures, all visual, auditory, and sensory data collected and processed by the robot to function as intended will be discarded once the procedures have been completed. The only exception is the recording of the number of interactions the robot has with each participant. However, these interactions will remain anonymous. All research data will be made openly available for secondary analysis 3 years after the project’s completion.

The data collected and processed by the robot is only stored locally (ie, within the robot itself) and is not transmitted over the internet or cloud services. Only the relevant data mentioned in the “Statistical analysis” section will be extracted from the log.

These data will be anonymous because it will be impossible to trace the identity of those involved in the interaction. The logs only contain information about the functions activated by the robot (eg, spoken phrases and gestures) and the phrases spoken by the user, along with the percentage of understanding achieved by the robot. The robots do not save any audio-video data. In the Noldus system, data are also saved and analyzed locally to avoid any internet or cloud sharing. Data imported into the system will be MP4 files from GoPro camera recordings. These files will be named using a code that identifies the user without revealing their identity (eg, “User_01” or “User_01_ddmmyyyy” to link to the date of interaction). Both the robot logs and the Noldus analyses will be saved on INRCA computers assigned to the Youse lab and protected by passwords, making them accessible only to the study’s research team.

Research data will remain available for secondary analyses for up to 3 years after study completion, as declared in the approved protocol.

#### Legal and Technical Aspects

The study will be conducted in accordance with all relevant regulatory and legal requirements and received approval from the Marche Territorial Ethics Committee (CET MARCHE).

Additionally, all potentially eligible participants must receive full information about the study and provide their consent to participate. Participants must also consent to the processing of their personal data in an anonymous and aggregated form, in accordance with EU Regulation 2016/679 (GDPR), the Italian Data Protection Code (APPI), and Legislative Decree No. 101/2018, which adapts national regulations to the European Regulation 2016/679. Participants must be informed that authorized personnel, members of the relevant ethics committee, and officials from the competent regulatory authorities may review their data. They will also be asked to provide specific informed consent to participate in the study, including consent for data retention for up to 3 years after the study’s completion.

All study information and consent forms will be explained verbally in plain, nontechnical language by a trained researcher. Also, flyers that present the project in an understandable language will be available at the pilot site. Moreover, participants may have a family member or caregiver present during the explanation if they wish. Only individuals who demonstrate understanding of the study and voluntarily agree will provide written informed consent.

Each signature must be dated by the signatory in person, and the informed consent form and any additional patient information must be retained by the investigator. Each patient will receive a signed copy of the informed consent form and the information sheet.

Participant information and consent forms are included in the documentation submitted to the local ethics committee for approval. No compensation was provided to participants.

Participants may opt to consent to the storage and use of their data long after the end of the project as part of open access to scientific publications and open research data, as required by the European Commission.

## Results

The recruitment started in January 2025 and will continue until December 2025. The recruited end users are reported in Table 1, grouped by phase of study. The assessment of the perceived quality and usability of the reception service and the analysis of HRI aim to provide an overall overview of the experience of older users with robots in a hospital setting.

**Table 1.** Results and clinical assessment.

Dimension	Scale	T0^a^	T1^b^
General information	Sociodemographic Questionnaire	✓	
Cognitive status	SPMSQ^c^	✓	
Functional status	Health Status Questionnaire	✓	
Usability	SUS^d^		✓
Perceived quality	Customer Satisfaction Questionnaire		✓
HRI^e^	Godspeed		✓
HRI	Noldus system		✓

a T0: recruitment.

b T1: assessment at the end of the interaction.

c SPMSQ: Short Portable Mental Status Questionnaire.

d SUS: system usability scale.

e HRI: human-robot interaction.

The SUS will be used to quantify the level of perceived usability for each of the 2 robotic platforms tested. The score obtained will also allow a direct comparison between the 2 systems, to explore potential differences in usability and user experience between Nao and Pepper, without being powered for definitive conclusions.

The Godspeed Questionnaire will be used to identify aspects related to the perception of robotic systems, such as the degree of anthropomorphism, animacy, likeability, perceived intelligence, and safety. These data may suggest useful ideas for optimizing the behavioral design of social robots.

Finally, the Noldus system will enable a dynamic analysis of the interaction to be conducted, based on objective and coded observation of the user behavior during interaction with the social robot. In addition to the temporal analysis of action sequences (eg, response times), the Noldus will also detect the emotions elicited during the interaction, thus providing additional qualitative elements useful for understanding the user’s subjective experience and the emotional impact of the interaction.

The feasibility of the HOSPER study will be evaluated and discussed in a separate publication expected to be published by 2027.

## Discussion

The HOSPER study addresses the feasibility of providing an innovative reception service within the IRCCS INRCA hospital in Ancona through social robots. This feasibility pilot is expected to show that a social robot–based reception service is acceptable to older adults accessing a geriatric hospital, with adequate recruitment, interaction completion, and questionnaire completion rates according to the predefined feasibility thresholds. It is hypothesized that the service will be associated with favorable satisfaction and usability profiles, suggesting potential to improve older adults’ reception experience, while also revealing technical and organizational constraints that must be addressed before routine implementation. These anticipated findings will primarily inform refinement of the service and the design of a subsequent, adequately powered trial, rather than demonstrating the effectiveness of the solution. A distinctive feature of the protocol is its person-centered design, which integrates standard usability measurements (ie, SUS) with robots’ logs and measures of participants’ subjective experience, including dynamic behavioral observation through the Noldus system.

Previous studies have shown that social assistive robots such as Pepper and Nao can support communication, emotional well-being, and engagement among older adults and other vulnerable groups, including in hospital and long-term care settings [13,27,29]. However, most prior work has focused on ward-based activities, companionship, or therapy, whereas HOSPER concentrates on the reception and orientation phase, which represents a critical aspect in the patient journey and has been less systematically explored [2,30]. Thus, this study extends existing evidence on social robotics in healthcare towards front-desk services in a geriatric hospital context.

The target population in this pilot was required to show full cognitive capacity to ensure participant safety and data quality during the first implementation of the robot-mediated reception service. This choice was intended to avoid both excessive frustration among participants and the introduction of major confounding factors in the interpretation of feasibility outcomes (eg, completion rates, usability, and satisfaction scores), but it also limits generalizability to older adults with cognitive impairment, who represent a substantial proportion of the geriatric population accessing the hospital. Once feasibility, usability, and basic reliability of the solution are established in this cognitively intact group, extending the interaction to older people with mild cognitive impairment will therefore be an important next step for future studies. Similarly, another limitation of the present study is the continuous presence of a dedicated researcher during robot interactions, which does not represent a real-world scenario. However, this controlled set-up is appropriate for early-stage feasibility and will inform how responsibilities could be progressively transferred to regular reception staff in later implementation phases, including training needs and procedures in case of technical failures. Further constraints arise from the need to operate the robots offline, which limits vocabulary size and dynamic updates, and from the controlled environment that does not fully reproduce the ambient noise and congestion of the hospital hall. In addition, potential resistance or skepticism from reception staff and clinicians, as well as unexpected breakdowns in robot functioning in a busy lobby, are anticipated challenges that will need to be monitored and addressed in future implementation phases through targeted training and clear repair procedures.

From an implementation science perspective, this feasibility pilot primarily focuses on intervention-level characteristics (usability, acceptability, and basic technical reliability) rather than a full evaluation of organizational context and economic impact. Therefore, only preliminary insights on the inner setting (eg, compatibility with current reception workflows and staff workload) will be obtained through the researcher’s observations, and no formal cost or cost-effectiveness analysis will be conducted at this stage. Future phases, informed by implementation frameworks, will need to explicitly examine organizational readiness, long-term integration into hospital processes, and the economic implications of scaling a robot-based reception service for administrators and policymakers [31-33].

## Abbreviations

GDPR: General Data Protection Regulation
HOSPER: analysis of the quality of reception services using robotic platforms, Nao and Pepper
HRI: human-robot interaction
IRCCS INRCA: Istituto Nazionale di Riposo e Cura per Anziani
SAR: socially assistive robot
SPSS: Statistical Package for the Social Sciences
SUS: system usability scale
